# Medico-Dental Education

**Published:** 1846-03

**Authors:** 


### Miscellaneous Notices.

*Medico-Dental Education.*—The importance of medico-dental education cannot be too strongly impressed upon the mind of the dental practitioner and student or individual, who may expect to engage in this department of practice. We mean that every dentist who is now in practice, or who hereafter expects to enter upon the practice, should combine the study of general medicine with his dental knowledge—embracing, at least, the study of anatomy, physiology and pathology.

Without this preparatory measure, we insist upon it, he cannot advance either the respectability of himself or permanently add to his own reputation, skill, or success, much less enhance the honor of the profession or contribute, in any very great measure, to relieving the sufferings of mankind, or protecting men from the wholesale quackery to which they are daily exposed.

Dr. Arnott very justly as well as beautifully remarks, that, "no man can well understand a subject of which he does not carry a distinct outline in his mind," both of the "synopsis, analysis, chapters and sections.

The synopsis gives a general view of the subject, like what a traveller obtains of a new country from a lofty central peak commanding the whole.

The analysis gives a view of one division like what the traveller has of a portion of the country from a lower summit."

While the chapters and sections direct attention to "single vallies or fields in the wide and beautiful domain of nature;" so with the study of dentistry, the student, if he wishes to obtain most complete mastery over his profession —to acquire the greatest amount of skill, success and reputation, to advance the dignity of his calling, and give the greatest possible security to the health and lives of his fellows.

To accomplish all this, we say it is indispensable that he should ascend this lofty peak and take a general view of the whole subject, as properly embraced and comprehended in the science and art of dental surgery, that then and not till then can he or should he be prepared to enter upon any smaller department of examination or practice; for, from this lower summit it is impossible he can view this smaller division in all its various relations, and, without which knowledge, he cannot understand it fully, much less can he practice either safely or successfully.

This leads us to notice an error, of no common magnitude among dentists, as well as among the people at large, to wit: that the teeth are viewed as so many foreign bodies, placed in the upper and lower jaws—that they have little or no connection with the rest of the system, and hence that any body, it matters very little whether he be a shoemaker or blacksmith, so that he can handle a tool, is sufficiently competent to extract a tooth. This may provoke a smile, but we are serious when we say that such a sentiment is by far too prevalent.

The object of these remarks is, if possible, to correct such a notion, and did time permit, it could very easily be shown that the teeth, instead of being foreign bodies and having no relationship with the rest of the system, have, on the contrary, the most direct, positive, intimate and inseparable connection with many, if not all the organs of the body.

Hence, we find and daily witness the closest sympathy of action and reaction between the teeth and the rest of the system.

Who has not seen the tender infant thrown into convulsions from teething? There is a relation between the fifth pair of nerves and the brain, and through the brain, with the muscular system. Anatomy and physiology discloses this fact.

Who does not know that *digestion* is impaired by decayed teeth? and the crowns of the teeth destroyed by faulty digestion? showing the connections between the dental organism and the stomach and intestines, liver, &c. &c.

This will suffice for the present to draw attention, and we hope sufficient attention, to this subject, that all, and especially dentists, will examine it closely, and we have no fear but that the result will be salutary, both in the elevation of the profession and the pulling down of quackery.

We are happy to say that many noble efforts have been made and are making, both by states and individuals, for the accomplishment of this praiseworthy object.

